# Delays in the diagnosis of six cancers: analysis of data from the *National Survey of NHS Patients*: *Cancer*

**DOI:** 10.1038/sj.bjc.6602587

**Published:** 2005-05-03

**Authors:** V L Allgar, R D Neal

**Affiliations:** 1 Centre for Research in Primary Care, University of Leeds, 71-75 Clarendon Road, Leeds LS2 9PL, UK; 2 Department of General Practice, Wales College of Medicine, Cardiff University, Wrexham Technology Park, Wrexham LL13 7YP, UK

**Keywords:** delay, diagnosis, patient, primary care, secondary care, referral

## Abstract

The aim of this paper is to describe and compare components of diagnostic delay (patient, primary care, referral, secondary care) for six cancers (breast, colorectal, lung, ovarian, prostate and non-Hodgkin's lymphoma), and to compare delays in patients who saw their GP prior to diagnosis with those who did not. Secondary data analysis of *The National Survey of NHS Patients*: *Cancer* was undertaken (65 192 patients). Breast cancer patients experienced the shortest total delays (mean 55.2 days), followed by lung (88.5), ovarian (90.3), non-Hodgkin's lymphoma (102.8), colorectal (125.7) and prostate (148.5). Trends were similar for all components of delay. Compared with patient and primary care delays, referral delays and secondary care delays were much shorter. Patients who saw their GP prior to diagnosis experienced considerably longer total diagnostic delays than those who did not. There were significant differences in all components of delay between the six cancers. Reducing diagnostic delays with the intention of increasing the proportion of early stage cancers may improve cancer survival in the UK, which is poorer than most other European countries. Interventions aimed at reducing patient and primary care delays need to be developed and their effect on diagnostic stage and psychological distress evaluated.

There is good evidence that UK patients' cancers are diagnosed at a more advanced stage compared with patients in other European countries (Berrino *et al*, 2001); this may partly explain the poorer survival of UK cancer patients. Reducing diagnostic delays may increase the proportion of early stage cancers and improve survival. Delays may occur at different stages of the cancer diagnostic journey: ‘patient delay’ (from onset of symptoms to their first presentation); ‘primary care delay’ (from first presentation in primary care to referral for further care or diagnostic investigation); ‘referral delay’ (from referral for further care or diagnostic investigation to being seen in secondary care); and ‘secondary care delay’ (from being first seen in secondary care to diagnosis). Patients diagnosed through screening bypass patient and primary care delays. Current UK policy aimed at earlier detection of cancer by reducing delays concentrates mainly on treatment and referral delays (DoH, 2000a, 2000b), although the evidence base for these policies has been questioned (Jones *et al*, 2001).

The literature regarding the length of diagnostic delays has several common themes. With the exception of cancer registry studies, most of the studies report conflicting findings from relatively small numbers of patients; generalisation from these data is difficult. This is compounded by different healthcare settings, different methods of measuring delays, the potential confounding effect of lead-time bias, and variations between cancers; and is reflected in the conflicting findings from this literature. Data from the literature relating to delays are shown in Table 1. The effect of delays on clinical outcomes varies between cancers. In breast cancer, delays of 3–6 months are associated with poorer survival (Richards *et al*, 1999), although some patients who present early may have poorer outcomes (Sainsbury *et al*, 1999). In colorectal cancer, delays have not been shown to have an effect on survival (Roncoroni *et al*, 1999), and although somewhat inconclusive, shorter delays have been associated with earlier stage diagnosis for rectal but not for colonic tumours (Arbman *et al*, 1996; Kiran and Glass, 2002). In lung cancer, there is some evidence that early stage disease has better survival (Mountain, 1997), although there is wide variation in the findings of reported studies (Jensen *et al* 2002; Moody *et al*, 2004; Myrdal *et al*, 2004). In ovarian cancer, there is no evidence that delays in referral or diagnosis affects survival at 18 months (Kirwan *et al*, 2002), although women with earlier stage disease are diagnosed faster (Wikborn *et al*, 1996). We are not aware of any studies that have examined the effect on diagnostic delay of seeing the GP prior to a cancer diagnosis compared with not seeing the GP (whether by a screening, or in-patient diagnosis following an emergency admission or via A&E). For some cancers, especially lung, morbidity and psychological outcomes may be more important than mortality. Psychological distress correlates positively with total diagnostic delay (Risberg *et al*, 1996), itself a reason to minimise delays.

The aim of this paper is to describe and compare the components of diagnostic delay (patient, primary care, referral, secondary care) for six types of cancer (breast, colorectal, lung, ovarian, prostate or non-Hodgkin's lymphoma), using patient-reported data from the *National Survey of NHS Patients: Cancer*. A secondary aim is to compare delays in those patients who reported seeing their GP prior to diagnosis with those who bypassed their GP, for whatever reason.

## MATERIALS AND METHODS

### Section 1 – National Survey of NHS Patients: cancer

The National Survey of NHS Patients: Cancer assessed quality of care of hospital patients in 172 Trusts in England (DoH, 2002). Patients with one of six types of cancer (breast, colorectal, lung, ovarian, prostate or non-Hodgkin's lymphoma) discharged from hospital between July 1999 and June 2000, participated in the survey. From a total sample of 123 984 patients, 92 683 were sent a questionnaire, after excluding those patients whose death had been registered. Completed questionnaires were returned by 65 337 patients. This represented a response rate of 74%, after allowing for returned mail, patients whose death had been reported after mailing and ineligible patients. In the sample, 65 192 were diagnosed with one of six types of cancer: female breast cancer (25 627), colorectal (15 891), prostate (10 992), non-Hodgkin's lymphoma (5604), lung (4011) and ovarian (3067). A total of 145 male patients with breast cancer also participated in the survey but were not included in this analysis because of their small numbers. The data set was obtained from the Data Archive at the University of Essex in 2003. Part D of the questionnaire asked a series of questions devoted to ‘finding out what was wrong with you’ (Box 1).

### Section 2 – Calculating components of delays, exclusions, and assumptions

**Figure 1:**
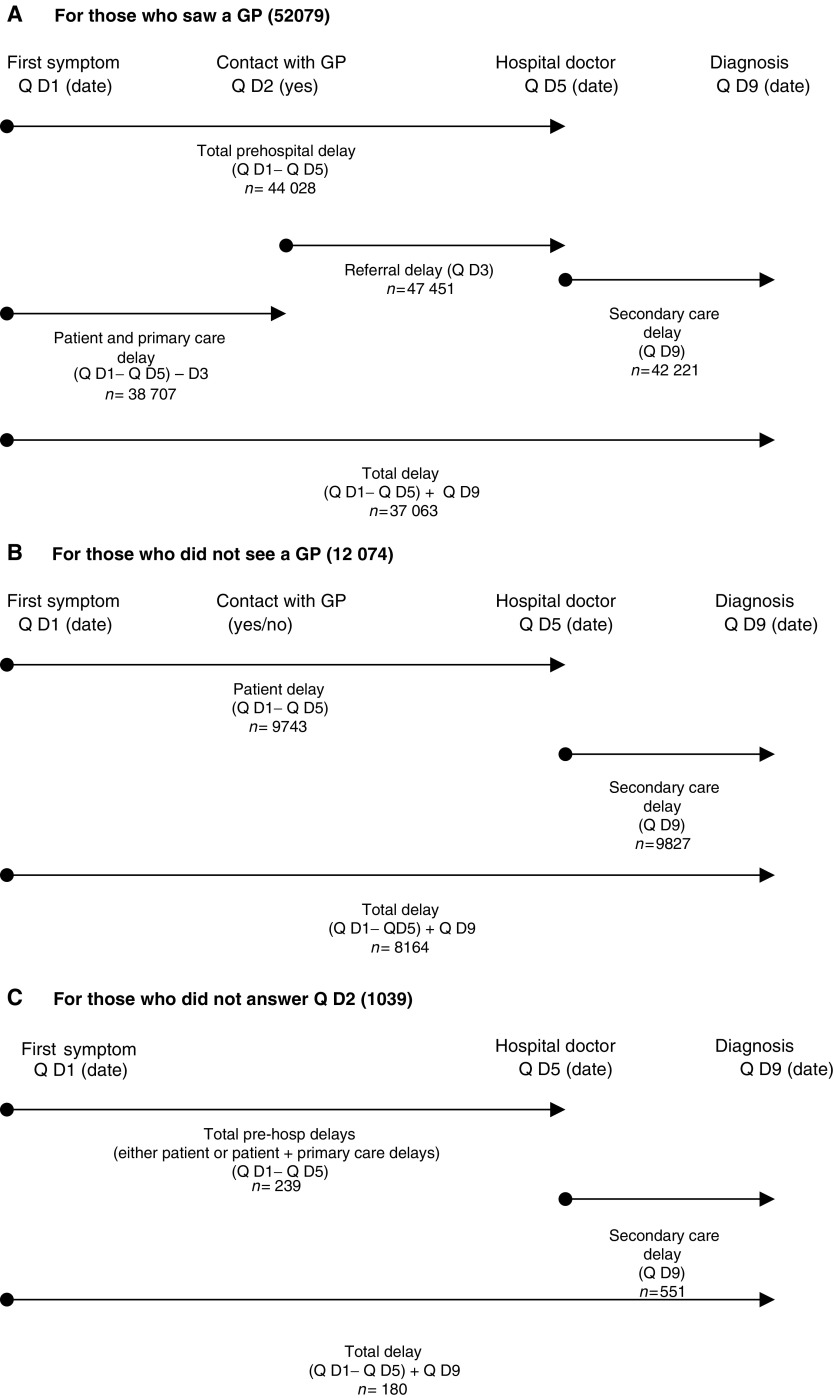
Individual components of delay in diagnosis.

Delays were calculated differently for patients who reported visiting their GP prior to diagnosis and those who had not, because of ways in which the questions were asked. This process is highlighted in Figure 1.

#### Total prehospital delay

This was calculated as the number of days between the date of appointment with hospital doctor (Q D5) and the date that symptoms were first noticed (Q D1). These were both asked as month and year. The first of the stated month was used for calculations. Total prehospital delays could not be calculated for patients whose first appointment with a hospital doctor was before the date they first noticed symptoms, or if one or both dates were missing. For those patients who did not see their GP prior to diagnosis, the ‘total prehospital delay’ is equivalent to ‘patient delay’ since there were neither primary care nor referral delays.

#### Referral delay

Referral delay was derived from Q D3, a categorical response variable, and was calculated by assigning a mid-point to the categories used (see Box 2). Referral delays could not be calculated for: patients who could not have had any referral delay because they did not see their GP prior to their diagnosis (i.e. answered no to Q D2, or did not answer Q D2); patients who had a delay of more than 1 year, since the categorisation did not permit calculation; and those who did not respond or could not remember.

### Patient and primary care delay

For patients who saw their GP prior to diagnosis, patient and primary care delays were calculated by subtracting the referral delay (as above), from the total prehospital delay (as above). Patient and primary care delays could not be calculated if the referral delay or the total prehospital delay was not available, or if the referral delay was longer than the prehospital delay, giving a false-negative patient and primary care delay. This arose because of the assumptions of mid-points and first of the month as described above.

#### Secondary care delay

This was calculated from question Q D9 (a categorical response variable), which asked when the patient found out their diagnosis. As with Q D3, the number of days was calculated by assigning a mid-point to the categories (Box 2). Secondary care delays could not be calculated for patients who responded ‘before first appointment’ or ‘more than 6 months’, or those who ‘had not been told’ or did not answer.

#### Total diagnostic delay

Total diagnostic delay was calculated by adding total prehospital delay to secondary care delay. As described above, this excludes patients who were diagnosed either before or more than 6 months after their first hospital appointment. Total delays could not be calculated if either secondary care delay or prehospital delay was missing.

### Section 3 – statistics

Diagnostic delay between the six cancer groups were compared using Kruskall–Wallis tests; χ^2^ tests were used for the categorical data. Mann–Whitney tests were used for comparisons within each cancer group between those patients who saw a GP and those who did not. A *P*-value of <0.05 was used to indicate statistical significance. All analyses were performed on SPSS (Version 11). The data are interval scale data; means and standard deviations (s.d.) are presented. However, due to the skewed nature of some of the distributions medians and interquartile ranges (IQR) are also presented for completeness. Confidence intervals around the means were calculated and are presented where appropriate in the figures. The large variation in delays resulted in some negative lower limits and large upper limits for some of the categories; these are not presented in the tables.

## RESULTS

### Numbers of patients seeing their GP prior to diagnosis

A total of 52 079 (80%) patients reported visiting their GP before their diagnosis, 12 074 (18%) reported that they had not, and 1039 (2%) did not answer this question. There was variation between the cancers, with 27% of breast cancer patients, but only 10% colorectal cancer patients, not seeing their GP prior to diagnosis (Table 2). In order to investigate this further, a sensitivity analysis was conducted, for breast cancer, to compare data for women within the screening age range (50–64) with women outside this age range. In all, 58% of the screening age women saw their GP, compared with 84% for those outside the screening age.

### Main findings

The numbers of patients for whom different components of delay were calculated are shown in Table 3. Table 4 shows the mean (s.d.) and median (IQR) for each component of diagnostic delay by cancer type. There were significant differences for all the components of delay between the six site-specific cancers (Table 5).

### 1. Total diagnostic delay

**Figure 2:**
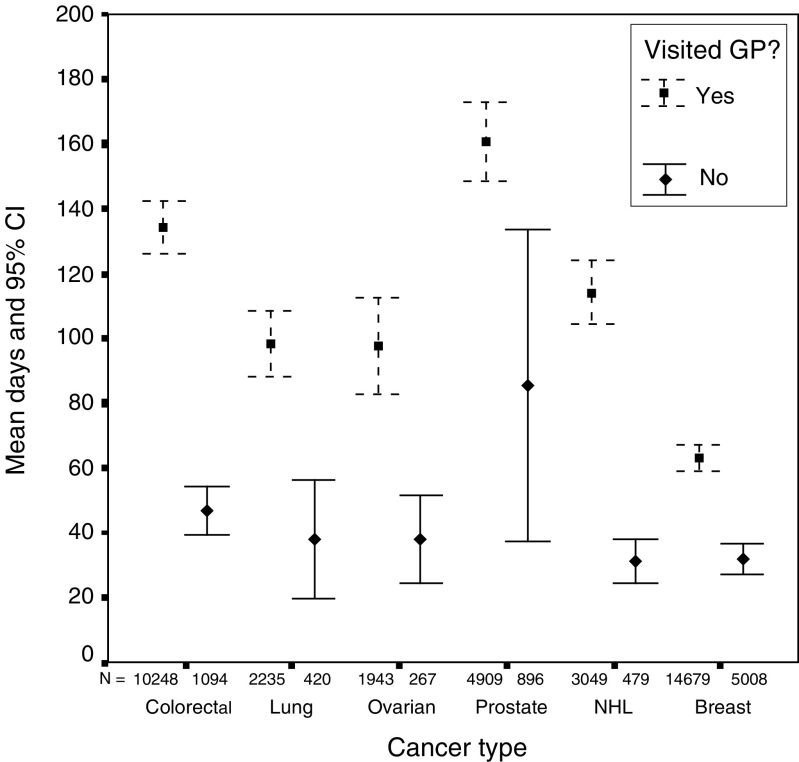
Total diagnostic delay (GP *vs* non-GP).

Breast cancer patients experienced the shortest mean and median delays, followed by lung, ovarian, non-Hodgkin's lymphoma, colorectal and prostate. Delays were considerably shorter in all cancers for those patients who did not report seeing their GP prior to diagnosis (Figure 2). There was a significant difference in total diagnostic delay between those patients who saw their GP and those who did not for colorectal (t(11 340)=6.9, *P*<0.001), lung (t(2653)=4.7, *P*<0.001), ovarian (t(2208)=2.9, *P*=0.004), prostate (t(5803)=4.2, *P*<0.001), non-Hodgkin's lymphoma (t(3526)=6.6, *P*<0.001) and breast (t(19 685)=7.9, *P*<0.001). In each case, those who saw their GP had a longer delay than those who did not.

### 2. Prehospital delays

Some patients in each cancer group reported no delay from first noticing symptoms until they saw a hospital doctor: breast 10 601 out of 22494 (47%), lung 1177 out of 3260 (36%), ovarian 932 out of 2673 (35%), non-Hodgkin's lymphoma 1628 out of 4650 (35%), prostate 2391 out of 7759 (31%), and colorectal 3467 out of 13 174 (26%). There was a significant difference between cancer groups (*χ*^2^(5)=1765.0, *P*<0.0001).

#### 2a. Total prehospital delays

**Figure 3:**
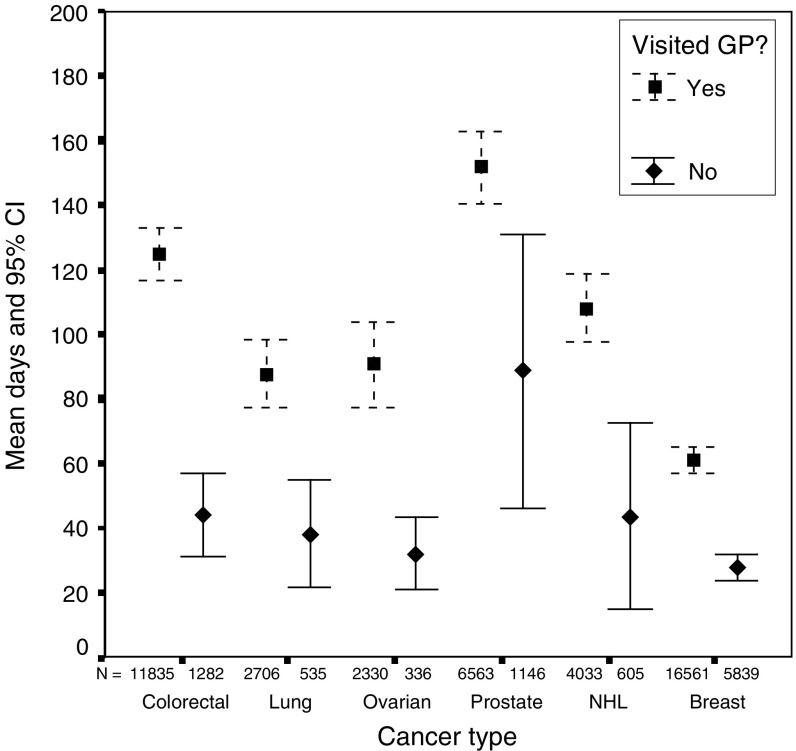
Total prehospital delay (patient, primary care and referral delay for GP patients and patient delay for non-GP patients).

The total prehospital delays were analysed by those who saw a GP prior to diagnosis and those who did not, since they are measuring different processes in each case (Figure 3). There was a significant difference in total prehospital delay between those patients who saw their GP and those who did not for all cancers: colorectal (t(13 114)=6.4, *P*<0.001); lung (t(3239)=3.9, *P*<0.001); ovarian (t(2664)=3.3, *P*=0.001); prostate (t(7707)=3.9, *P*<0.001); non-Hodgkin's lymphoma (t(4636)=4.3, *P*<0.001); and breast (t(22 398)=8.7, *P*<0.001). In each case, those who saw their GP had a longer delay than those who did not.

#### 2b. Patient and primary care delays

**Figure 4:**
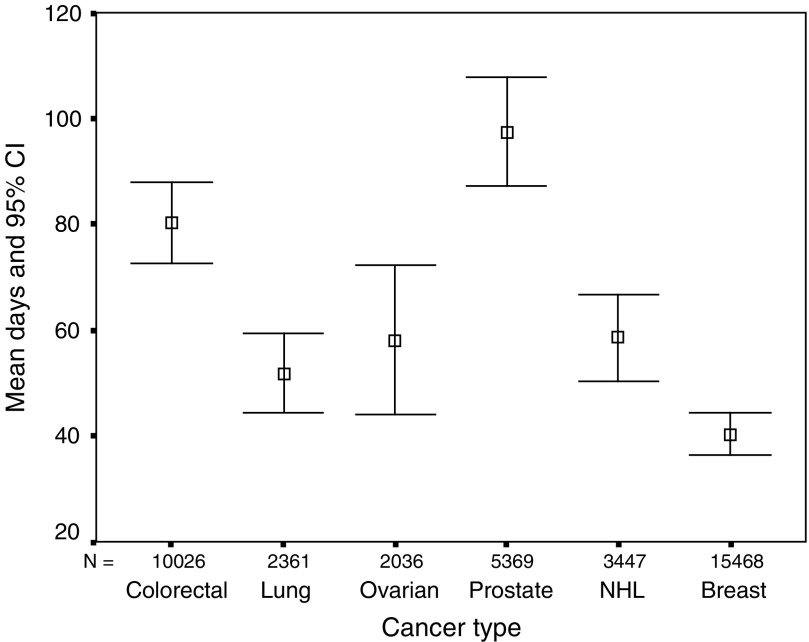
Patient and primary care delay.

The shortest delays were experienced by patients with breast cancer, followed by lung, ovarian, non-Hodgkin's lymphoma, colorectal and prostate (Figure 4).

#### 2c. Referral delays

**Figure 5:**
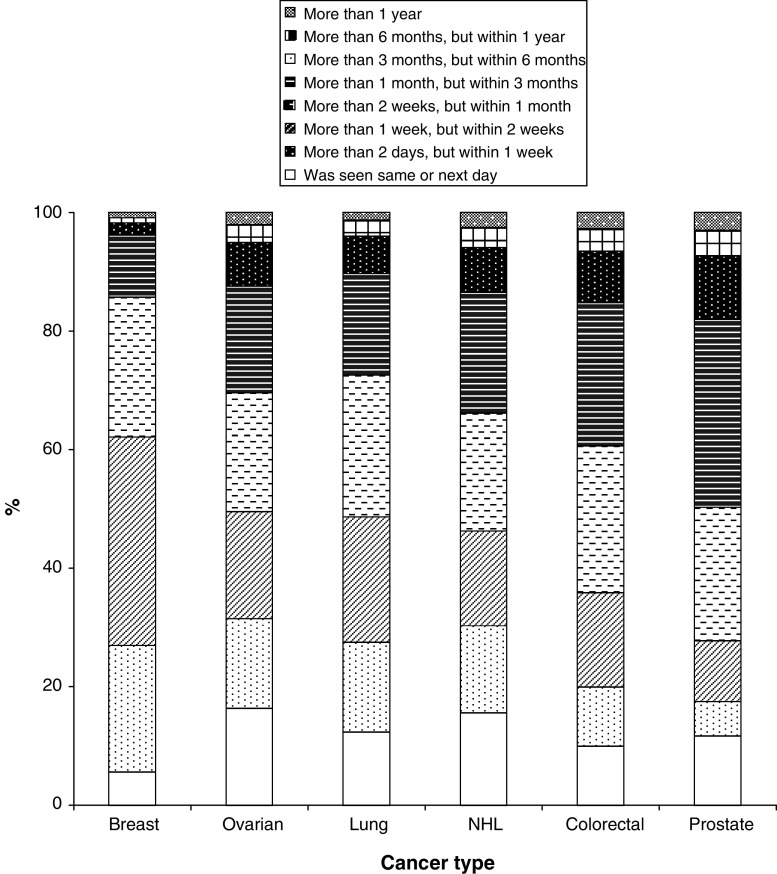
Referral delay.

The shortest delays were experienced by patients with breast cancer, followed by lung, ovarian, non-Hodgkin's lymphoma, colorectal and prostate (Figure 5). Over 60% patients with breast cancer were seen within 2 weeks, compared with less than 30% prostate cancer patients.

### 3. Secondary care delay

**Figure 6:**
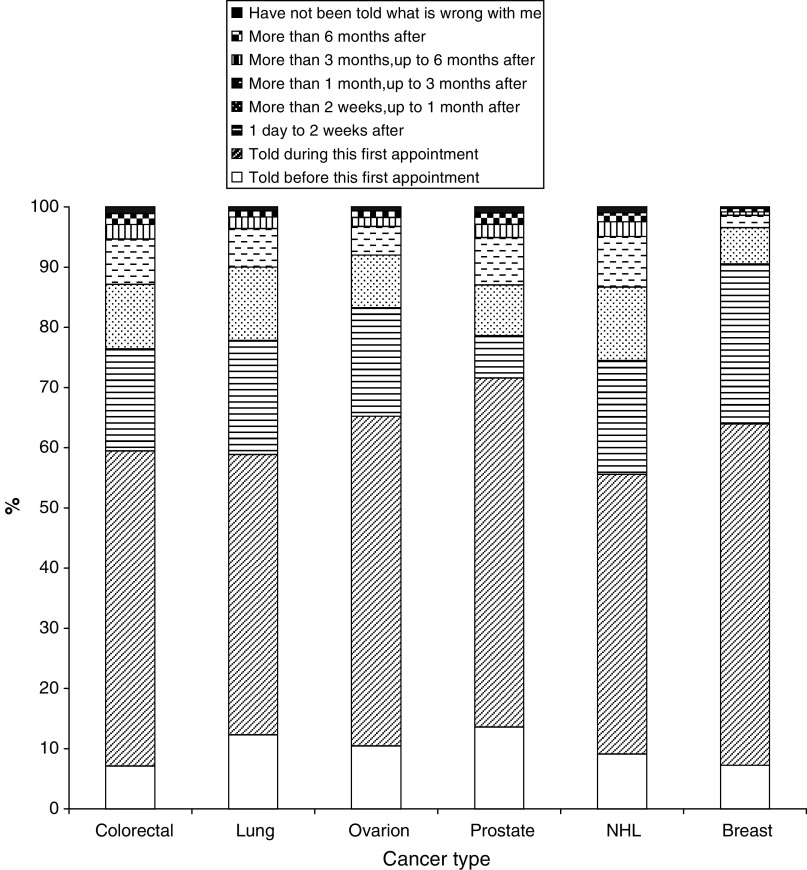
Secondary care delay.

**Figure 7:**
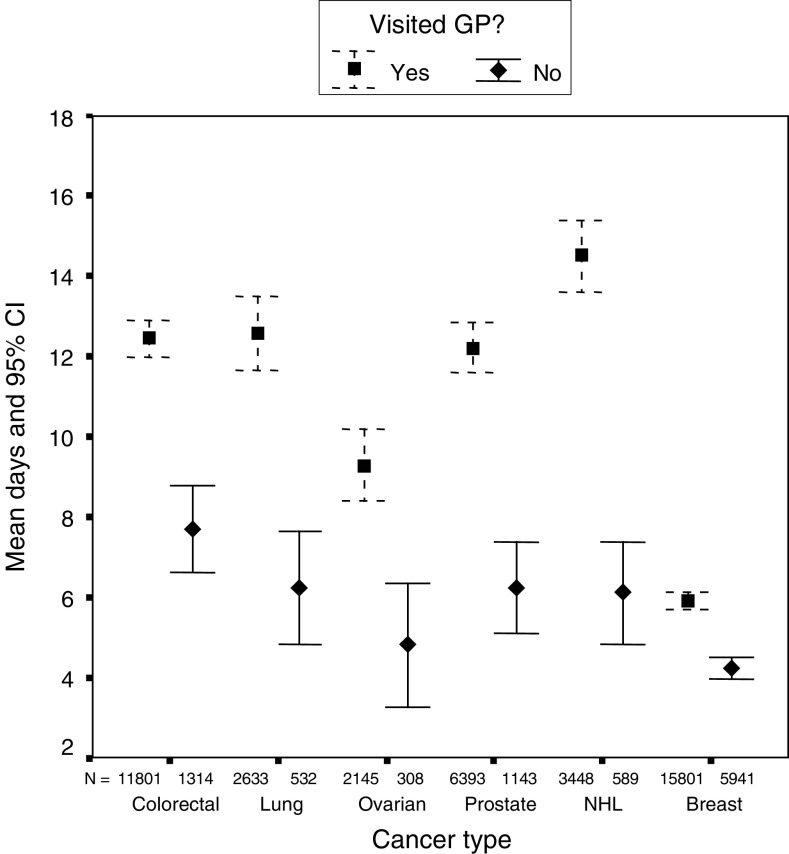
Secondary care delay by GP contact.

These delays were considerably shorter than referral and prehospital delays (Figures 6 and 7). In all 9% found out their diagnosis prior to their first hospital appointment, but this varied significantly between cancer groups. Delays were shortest for breast cancer, followed by ovarian, prostate, lung, colorectal and non-Hodgkin's lymphoma. The median number of days was zero for all six cancer groups; the majority of patients found out their diagnosis at their first hospital appointment. Patients who saw their GPs prior to diagnosis had longer secondary care delays than those who did not. There was a significant difference for each cancer group: colorectal (t(13 113)=6.6, *P*<0.001); lung (t(3163)=5.8, *P*<0.001); ovarian (t(2451)=3.6, *P*<0.001); prostate (t(7534)=7.5, *P*<0.001); non-Hodgkin's lymphoma (t(4035)=7.5, *P*<0.001); and breast (t(21740)=8.2, *P*<0.001). In each case, those who saw their GP had a longer delay than those who did not.

### Assessing the effect of assumptions made in main data analysis

Referral delay was greater than 1 year for 2% of the sample; these were excluded from the main analysis. Similarly, 1% of patients with a secondary care delay of greater than 6 months were excluded from the main analysis. In order to determine the effect of this on the analysis, we reanalysed the data, coding delays of greater than 1 year as 365 days and delays of 6 months as 168 days. This showed that the mean delay increased, as would be expected, but that the median delays remained the same for all except referral delay for ovarian cancer, where there was an increase from 11 days to 21 days (Table 6).

## DISCUSSION

### Statement of principal findings

This paper reports findings from the analysis of a large data set of patients relating to their cancer diagnosis, and is the largest single comprehensive study of diagnostic delays in cancer. It is one of few studies to report delays in prostate cancer and the first to do so in non-Hodgkin's lymphoma. Total diagnostic delays remain long, particularly in some cancers. Breast cancer patients had the shortest delays compared with the other cancers. The mean delay of 8 weeks still suggests that the diagnostic process could be faster. Prostate cancer had the longest delays compared with the other cancers. Of more concern are the lengthy delays for colorectal cancer. Patients with lung cancer, ovarian cancer and non-Hodgkin's lymphoma also experienced considerable delays. Patient and primary care delays contributed to larger proportions of the total diagnostic delay than did referral delays and secondary care delays. Over a third of the sample reported no prehospital delays, either because there were no delays (i.e. cancer found by screening, or asymptomatic tumours found while under investigation for other problems) or because the symptom and the first hospital visit occurred within the same month. Large differences were found in all components of delay between patients who reported seeing their GP prior to diagnosis and those who did not (diagnosed through screening or diagnosed while an in-patient) with patients who reported seeing their GP experiencing longer delays.

#### Strengths and weaknesses of the study

All self-report questionnaires have to be interpreted with some caution. Sampling in 172 NHS Trusts in England ensured generalisability. There is the potential for bias in that different proportions of patients between the six cancer groups would have died prior to questionnaire administration; furthermore, these patients are likely to have had more aggressive disease and to have presented differently to those who survived. There is the potential for recall bias, especially given the time interval between diagnosis and survey completion, for at least some of the sample. Data were not collected relating to diagnostic stage, comorbidity, histological type of cancer, or the natural history of the cancer; hence, we cannot be sure that the sample was representative of the ‘cancer population’, and cannot exclude the possibility of confounding as a result.

The ways in which questions were asked forced us to make assumptions about the data; these may have affected some of the analyses. For example, time was calculated by using the first of each month as the reference date for ‘MM/YY’ variables. If patients experienced symptoms during the same month that they were first seen at the hospital, this time duration would have been recorded as zero. However, this should give rise to a neutral effect because patients who experienced symptoms at the end of a month and were seen at the hospital at the beginning of the next month would have a time duration recorded as 2 months. As a result of ‘open-ended’ time categories, patients with referral delays of greater than 1 year and with secondary care delays greater than 6 months were excluded. This only affected a small number of patients and did not influence the main findings. There may be some differences between the actual date of ‘tissue diagnosis’ and the date perceived by the patients as the date that they were told ‘what was wrong with them’. However, this would have been consistent across the data set. Our analysis divided patients who had reported seeing their GP for ‘this condition’ prior to diagnosis with those who did not. The survey did not ask patients about alternative routes into diagnosis, we are not therefore able to comment further on this. Lastly, the survey did not ask questions in such a way to permit patient and primary care delays to be calculated separately.

#### Strengths and weaknesses in relation to other studies, discussing important differences in results

Compared with previous studies of diagnostic delay in cancer, this paper reports data from a very large data set. There is no standard tool for asking patients about their delays; hence, comparisons between all such studies must be undertaken with caution. In breast cancer, our data show that delays were longer than most previously published reports. For prostate cancer, there are no previous data with which to compare total delays, but our data on referral delays are in keeping with previous data. For colorectal cancer, our figures show that referral delays were longer than other published data, but secondary care delays were shorter. Our figures for lung cancer compare favourably with the literature, with total delays, referral delays and secondary care delays all being shorter. Our figures for referral delay in ovarian cancer show slightly longer referral delays. We are not aware of any other work that has compared delays between patients who saw their GP prior to diagnosis, with those diagnosed via alternative pathways.

#### Meaning of the study: possible explanations and implications for clinicians and policymakers

Shorter delays in breast cancer compared with other cancers may occur because of a more straightforward presentation of signs and symptoms that are easily understood by patients and doctors, clear referral guidance, well-organised secondary care clinics, a national screening programme and a high public profile. Reductions in delays may improve survival. Longer delays in prostate cancer may occur because of the insidious onset of nonspecific symptoms, which may occur on top of preexisting urinary outflow symptoms. Delays may improve in the future with more opportunistic screening, although the effect of this on survival is unknown. The long delays in colorectal cancer may be for similar reasons; again the effects of the introduction of a national screening programme are unknown. While there is insufficient evidence at present to prove that shorter delays are associated with better prognosis, there is considerable logic that this should be the case, given the potential for curative treatments. The effects of these delays for lung cancer, ovarian cancer and non-Hodgkin's lymphoma are unclear. There is clear potential to reduce delays with the anticipated outcome of improved survival.

The finding that patient and primary care delays were the longest suggests that while further reductions in referral delays and secondary care delays may result in better psychological outcomes, attempts to improve clinical outcomes (earlier stage diagnosis and improved survival), must be directed at patient and/or primary care delays.

The percentage of patients with cancer who bypassed their GP in their diagnostic journey varied considerably between the six cancers. This may have been for one of two reasons. First, because of diagnosis by screening. From the literature, it would be expected that a significant numbers of breast cancers would be screen-detected (Banks *et al*, 2004). Our sensitivity analysis showed that for the female population in the age range for breast cancer screening, fewer patients saw their GP prior to diagnosis than those outside the age range, suggesting that screen-detected cancers were responsible for many of those apparently bypassing the GP. Second, through secondary care diagnosis following an emergency admission, by self-presentation to A&E or via an interspecialty referral. Such patients are likely to have varied presentations of often very advanced or rapidly progressing (and symptomatic) disease, and may, to a degree, confound the findings.

The finding that patients who reported seeing their GP prior to diagnosis had longer delays than those who did not was an unexpected finding. Given that the referral delays were not prolonged, there are two potential explanations of this finding. Firstly, because earlier stage disease (usually less symptomatic) presents mainly to primary rather than secondary care, with later stage disease (with more aggressive symptoms) more likely to be presented to secondary care. Second, because there may be more ‘system’ delays in primary compared to secondary care (e.g. waiting times for primary care initiated diagnostics). Shorter secondary care delays in patients not seeing their GP prior to diagnosis are probably explained by quicker access to diagnostic tests for in-patients compared with outpatients. The effect of these longer diagnostic delays in patients seeing their GP prior to diagnosis on stage at diagnosis and survival remains unknown. The differences in delays by diagnostic pathway is an important finding and needs further work.

The implications of these findings, the methodological limitations notwithstanding are that there are significant opportunities to reduce diagnostic delay in order to potentially improve clinical outcomes, at least for at-risk groups for some of these six cancers, and to potentially reduce psychological distress caused by delays (Risberg *et al*, 1996). This is in keeping with the recent National Audit Office report recommendation to tackle diagnostic delays (National Audit Office, 2004). However, such interventions must be considered within the context of the overall presentation of suspicious symptoms in primary care, and the low positive predictive symptoms of suspected symptoms, and the processes of diagnostic reasoning (including watchful waiting) and appropriate thresholds for referral as a result. Our findings will provide a baseline for comparison of future surveys to measure progress in reducing diagnostic delays.

#### Unanswered questions and future research

Prior to the development and evaluation of interventions to reduce delay (Jensen *et al*, 2002), further work needs to be performed in order to elucidate the separate contributions of patient and primary care delays to the overall delays. There may be variation between delays and socio-demographic factors, and local or regional variations; these need quantifying prior to intervention. Findings from the ever-increasing evidence base on the reasons for patient delays in most cancers, and the smaller evidence base regarding primary care delays (Spellman *et al*, 1999) will inform the development of the interventions. Lastly, work is needed to further explore the reasons for and implications of longer delays in patients who reported seeing see their GP prior to diagnosis, compared with those who did not.

## Figures and Tables

**Table 1:** Diagnostic delays in six cancers – summary of findings from the literature

	**Total delays**	**Patient delays**	**Primary care delays**	**Referral delays**	**Secondary care delays**	**Other**
Breast	15 days (Arndt *et al*, 2003)	0 days (Jones *et al*, 1992)4 weeks (Thongsuksai *et al*, 2000)1/4 >12 weeks (Thongsuksai, 2000)1 Week (Thulesius *et al*, 2004)14 days (Meechan *et al*, 2002)16 days (Arndt *et al*, 2002)13 days (Nosarti *et al*, 2000)	—	13 days (Sainsbury *et al*, 1999)11 days (Jones *et al*, 1992)9 days (urgent referrals) (Spurgeon *et al*, 2000)14 days (nonurgent referrals) (Spurgeon *et al*, 2000)13 days (Nosarti *et al*, 2000)	—	1/3 experience total pre-hospital delays of >12 weeks (Richards *et al*, 1999)Provider delay of 1 Week (Thulesius *et al*, 2004)
						
Colorectal	-	60 days (Aithal and Tanner, 1996)27.5 days (Thongsuksai *et al*, 2000)>6 weeks (54%) (Robinson *et al*, 1986)16.2 days (mean, rectum) (Holliday and Hardcastle, 1979)12.7 days (mean, colon) (Holliday and Hardcastle, 1979)20–66%) >3 months (Graffner and Olsson, 1986)	47%>6 weeks (Robinson *et al*, 1986)15.3 days (mean, rectum) (Holliday and Hardcastle, 1979)11.2 days (mean, colon) (Holliday and Hardcastle, 1979)20–34% >3 months (Holliday and Hardcastle, 1979)	16 days (Aithal and Tanner, 1996)11 days (Jones *et al*, 1992)13 days (urgent referrals) (Spurgeon *et al*, 2000)27 days (nonurgent referrals) (Spurgeon *et al*, 2000)	15 days (Aithal and Tanner, 1996)17 days (mean 52) (Potter and Wilson, 1999)	Mean delay to treatment delay: 244 days (Rectal), 149 days (colon) (Langenbach *et al*, 2003)
						
Lung	4.6 months (mean 5.8) (Myrdal *et al*, 2004)189 days (Koyi *et al*, 2002)109 days (Billing and Wells, 1996)	21 days (mean 43) (Koyi *et al*, 2002)31 days (Jones *et al*, 1992)	33 days (mean 56) (Koyi *et al*, 2002)	7 days (Jones *et al*, 1992)7 days (urgent referrals) (Spurgeon *et al*, 2000)12 days (nonurgent referrals) (Spurgeon *et al*, 2000)	9 days (mean 33) (Koyi *et al*, 2002)1.6 months (Myrdal *et al*, 2004)	19 (8.6%) total delay >1 year (of whom 18 had adenocarcinoma) (Yoshimoto *et al*, 2002)
						
Ovarian	55% <3 months, 26% >6 months, and 11% >12 months (Goff *et al*, 2000)56% <4 weeks, 30% >8 weeks, and 4% <4 days (Wikborn *et al*, 1996)	78% <4 weeks (Kirwan *et al*, 2002)3.5 weeks (Thulesius *et al*, 2004)	73% <4 weeks (Kirwan *et al*, 2002)	6 days (Kirwan *et al*, 2002) 70% <2 weeks (Kirwan *et al*, 2002)6 days (urgent referrals) (Spurgeon *et al*, 2000)24 days (nonurgent referrals) (Spurgeon *et al*, 2000)	—	Mean total pre-hospital delay 95.3 days (s.d. 15.1 days) (Kirwan *et al*, 2002)Provider delay of 3.5 weeks (Thulesius *et al*, 2004)
						
Prostate	—	20 days (Jones *et al*, 1992)	—	15 days (Jones *et al*, 1992)19 days (urgent referrals) (Spurgeon *et al*, 2000)41 days (nonurgent referrals) (Spurgeon *et al*, 2000)	—	
						
NHL	—	—	—	—	—	

All delays are medians unless other stated.

**Table 2:** Number and percentage of patients seeing their GP prior to attending hospital

	**Colorectal**		**Lung**		**Ovarian**		**Prostate**		**Non-Hodgkin's lymphoma**		**Breast**		**Total**	
	***n***	**%**	***n***	**%**	***n***	**%**	***n***	**%**	***n***	**%**	***n***	**%**	***n***	**%**
Did see GP	14 032	88	3246	81	2638	86	9159	83	4742	85	18 262	71	52 079	80
Did not see GP	1612	10	694	17	395	13	1560	14	791	14	7022	27	12 074	19
Not answered	247	2	71	2	34	1	273	3	71	1	343	1	1039	2
														
Total	15 891		4011		3067		10 992		5604		25 627		65 192	

**Table 3:** Summary of the number of patients excluded, and those available for analysis for each component of diagnostic delay, by cancer type

	**Colorectal**	**Lung**	**Ovarian**	**Prostate**	**NHL**	**Breast**	**Total**
Number in the sample	15 891	4011	3067	10 992	5604	25 627	65 192
Total delay	11 385 (72%)	2669 (67%)	2216 (72%)	5840 (53%)	3537 (63%)	19 760 (77%)	45 407 (70%)
Secondary delay or total prehospital delay missing	4506	1342	851	5152	2067	5867	19 785
	15 891	4011	3067	10 992	5604	25 627	65 192
							
Total delay (if ‘yes’ to Q D2)	10 248	2235	1943	4909	3049	14 679	37 063
Secondary delay or total pre-hospital delay missing	3784	1011	695	4250	1693	3583	15 016
	14 032	3246	2638	9159	4742	18 262	52 079
							
Total delay (if ‘no’ to Q D2)	1094	420	267	896	479	5008	8164
Secondary delay or total pre-hospital delay missing	518	274	128	664	312	2014	3910
	1612	694	395	1560	791	7022	12 074
							
Total delay (if no response to Q D2)	43	14	6	35	9	73	180
Secondary delay or total pre-hospital delay missing	204	57	28	238	62	270	859
	247	71	34	273	71	343	1039
							
Total pre-hospital delay	13 174 (83%)	3260 (81%)	2673 (87%)	7759 (71%)	4650 (83%)	22 494 (88%)	54 010 (83%)
First appointment before had symptoms	453	158	65	382	168	507	1733
One or both dates missing	2264	593	329	2851	786	2626	9449
	15 891	4011	3067	10 992	5604	25 627	65 192
							
Total pre-hospital delay (if ‘yes’ to Q D2)	11 835	2706	2330	6563	4033	16 561	44 028
First appointment before had symptoms	388	134	53	320	112	319	1326
One or both dates missing	1809	406	255	2276	597	1382	6725
	14 032	3246	2638	9159	4742	18 262	52 079
							
Patient delay (if ‘no’ to Q D2)	1282	535	336	1146	605	5839	9743
One or both dates missing	275	135	48	354	130	999	1941
First appointment before had symptoms	55	24	11	60	56	184	390
	1612	694	395	1560	791	7022	12 074
							
Total prehospital delay (if no response to Q D2)	57	19	7	50	12	94	239
First appointment before had symptoms	10	0	1	2	0	4	17
One or both dates missing	180	52	26	221	59	245	783
	247	71	34	273	71	343	1039
							
Patient and primary care delay (if ‘yes’ to Q D2)	10 026	2361	2036	5369	3447	15 468	38 707
Referral delay or total prehospital delay missing	2959	675	407	3065	951	2076	10 133
False negatives	1047	210	195	725	344	718	3239
	14 032	3246	2638	9159	4742	18 262	52 079
							
Referral delay	12 527 (79%)	2950 (74%)	2453 (80%)	7877 (72%)	4242 (76%)	17 402 (68%)	47 451 (73%)
Answer ‘no’ to Q D2	1612	694	395	1560	791	7022	12 074
No response to Q D2	247	71	34	273	71	343	1039
Can't remember (Q D3)	777	186	78	838	285	474	2638
Not answered (Q D3)	371	71	56	200	104	227	1029
> 1 year (Q D3)	357	39	51	244	111	159	961
	15 891	4011	3067	10 992	5604	25 627	65 192
							
Secondary care delay	13 244 (83%)	3199 (80%)	2474 (81%)	7671 (70%)	4073 (73%)	21 938 (86%)	52 599 (81%)
Not answered	690	159	112	606	242	622	2431
Not told	166	26	20	95	47	68	422
> 6 months	266	38	30	173	68	137	712
Told before	1048	457	295	1251	420	1729	5200
NA	477	132	136	1196	754	1133	3828
Total	15 891	4011	3067	10 992	5604	25 627	65 192
							
Secondary care delay (if ‘yes’ to Q D2)	11 801	2633	2145	6393	3448	15 801	42 221
Not answered	505	95	83	390	164	385	1622
Not told	147	20	19	69	43	47	345
> 6 months	251	34	29	151	64	115	644
Told before	906	348	244	1112	343	981	3934
NA	422	116	118	1044	680	933	3313
Total	14 032	3246	2638	9159	4742	18 262	52 079
							
Secondary care delay (if ‘no’ to Q D2)	1314	532	308	1143	589	5941	9827
Not answered	90	39	19	115	51	134	448
Not told	18	6	1	24	4	19	72
> 6 months	12	4	1	21	4	21	63
Told before	133	98	48	119	72	722	1192
NA	45	15	18	138	71	185	472
Total	1612	694	395	1560	791	7022	12 074
							
Secondary care delay (if no response to Q D2)	129	34	21	135	36	196	551
Not answered	95	25	10	101	27	103	361
Not told	1	0	0	2	0	2	5
> 6 months	3	0	0	1	0	1	5
Told before	9	11	3	20	5	26	74
NA	10	1	0	14	3	15	43
Total	247	71	34	273	71	343	1039

**Table 4:** Summary of the components of diagnostic delay by cancer type (all data in days)

	**Colorectal**	**Lung**	**Ovarian**	**Prostate**	**NHL**	**Breast**
(1) Total delay						
All patients						
Mean (s.d.)	125.7 (395.2)	88.5 (239.8)	90.3 (320.0)	148.5 (494.3)	102.8 (256.7)	55.2 (241.8)
Median (IQR)	61 (29–143)	38 (7–91)	37 (7–92)	61 (7–126)	51 (7–117)	30 (0–38)
GP patients						
Mean (s.d.)	134.4 (413.5)	98.3 (247.5)	97.7 (338.5)	160.5 (437.7)	114 (273.2)	63 (258.9)
Median (IQR)	67 (30–151)	51 (21–99)	50 (7–99)	61 (30–150.5)	56 (21–122)	31 (0–56)
Non-GP patients						
Mean (s.d.)	46.9 (124.5)	38.1 (190.5)	37.9 (113)	85.6 (733.5)	31.2 (74.8)	31.8 (176.9)
Median (IQR)	7 (0–52)	0 (0–31)	7 (0–31)	0 (0–31)	0 (0–31)	7 (0–31)
No response to D2						
Mean (s.d.)	62.6 (103.3)	31.4 (47.8)	20.5 (37.2)	75.1 (99.6)	98.2 (136.8)	76.4 (387.7)
Median (IQR)	21 (0–86)	7 (0–45)	0 (0–46.3)	31 (0–122)	7 (0–180.5)	7 (0–31)
						
(2a) Total prehospital delay						
All patients						
Mean (s.d.)	116.7 (424.9)	79.1 (264.8)	83.1 (306.6)	141.7 (505.5)	99.7 (344.8)	52.2 (249.5)
Median (IQR)	59 (0–122)	31 (0–62)	31 (0–91)	31 (0–121)	31 (0–92)	29 (0–31)
GP patients						
Mean (s.d.)	124.8 (440.8)	87.6 (276.9)	90.7 (325.4)	151.5 (456.4)	107.9 (341.6)	60.8 (271.5)
Median (IQR)	61 (30–122)	31 (0–91)	31 (0–92)	61 (29–122)	31 (0–92)	30 (0–31)
Non-GP patients (=patient delay)						
Mean (s.d.)	44.3 (234.5)	38.2 (193.6)	32.1 (102.4)	88.5 (730.2)	43.7 (361.5)	27.7 (167.5)
Median (IQR)	0 (0–31)	0 (0–30)	0 (0–31)	0 (0–30)	0 (0–29)	0 (0–30)
						
(2b) Patients & primary care delay (GP patients)						
Mean (s.d.)	80.4 (393.9)	51.8 (187.1)	58.1 (327.9)	97.5 (379.2)	58.6 (244.5)	40.4 (245.9)
Median (IQR)	20 (0–66)	10 (0–40)	10 (0–40)	10 (0–66)	10 (0–40)	9 (0–20)
						
(2c) Referral delay (GP patients)						
Mean (s.d.)	42.8 (54.7)	33.1 (48.6)	34.8 (51.4)	50.0 (57.1)	37.2 (53.1)	20.8 (30.7)
Median (IQR)	21 (11–56)	21 (5–56)	11 (5–56)	21 (11–56)	21 (5–56)	11 (5–21)
						
(3) Secondary care delay						
All patients						
Mean (s.d.)	11.9 (24.7)	11.5 (23.0)	8.7 (20.4)	11.3 (25.2)	13.2 (25.2)	5.4 (13.5)
Median (IQR)	0 (0–7)	0 (0–7)	0 (0–7)	0 (0–7)	0 (0–21)	0 (0–7)
GP patients						
Mean (s.d.)	12.4 (24.2)	12.6 (23.9)	9.3 (21.2)	12.2 (25.9)	14.5 (26.4)	5.9 (14.3)
Median (IQR)	0 (0–7)	0 (0–21)	0 (0–7)	0 (0–7)	0 (0–21)	0 (0–7)
Non-GP patients						
Mean (s.d.)	7.7 (20.4)	6.3 (16.5)	4.8 (13.8)	6.2 (19.6)	6.1 (15.8)	4.2 (10.4)
Median (IQR)	0 (0–7)	0 (0–7)	0 (0–7)	0 (0–0)	0 (0–7)	0 (0–7)
No-response D2						
Mean (s.d.)	6.4 (14.3)	7.8 (23.6)	6.7 (17.1)	10.5 (27.2)	5.6 (13.8)	5.3 (17.9)
Median (IQR)	0 (0–7)	0 (0 – 2)	0 (0–0)	0 (0–7)	0 (0–5)	0 (0–0)

**Table 5:** Kruskall–Wallis statistical tests comparing the six cancer groups

**Delay type**	***χ* ^2^**	**df**	***P*-value**
Total prehospital delay	3859.9	5	<0.001
Patient, primary care and referral delay (GP patients only)	3172.5	5	<0.001
Patient delay (non-GP patients only)	17.2	5	0.004
Patient and primary care delay (GP patients only)	1490.4	5	<0.001
Referral delay (GP patients only)	2910.0	5	<0.001
Secondary care delay	572.9	5	<0.001
			
Total delay	3632.0	5	<0.001

**Table 6:** Mean (s.d.) and median (IQR) delays after recoding referral and secondary care delay

	***N***	**Number recoded**	**% *N***	**Mean (s.d.)**	**Median (IQR)**
*Referral delay*					
Colorectal	12 884	357	3	51.7 (75.5)	21 (11.56)
Lung	2989	39	1	37.4 (61.2)	21 (5.56)
Ovarian	2504	51	2	41.5 (69.0)	21 (5.56)
Prostate	8121	244	3	59.4 (77.8)	21 (11.56)
NHL	4353	111	3	45.5 (73.5)	21 (5.56)
Breast	17 561	159	1	23.9 (44.6)	11 (5.21)
					
*Secondary care delay*					
Colorectal	13 510	266	2	14.9 (32.7)	0 (0.7)
Lung	3237	38	1	13.3 (28.3)	0 (0.7)
Ovarian	2504	30	1	10.6 (26.7)	0 (0.7)
Prostate	7844	173	2	14.7 (33.8)	0 (0.7)
NHL	4141	68	2	15.7 (31.8)	0 (0.21)
Breast	22 075	137	1	6.44 (18.5)	0 (0.7)

**Box 1:** Questions analysed in this paper

• Q D1: When did you first notice signs or symptoms? (month and year)
• Q D2: Had you visited a GP about this condition before you attended hospital? (Yes/No)
• Q D3: (if answered Yes to D2) After visiting the GP, how long did you have to wait before your first appointment with a hospital doctor (predetermined categories, including cannot remember)?
• Q D5: When did you first see a hospital doctor for your condition? (month and year)
• Q D9: Were you told what was wrong with you during this first hospital appointment, or was it before or after this first hospital appointment? If told after this appointment, then when? (predetermined categories including NA)?

**Box 2:** Mid-point calculations for categorical variables

Referral delay		Secondary care delay	
Category	Mid-point number of days	Category	Mid-point number of days
Same or next day	1 day	Before first appointment	Unable to estimate
2 days–1 week	5 days	Told at first appointment	0 days
1–2 weeks	11 days	1 day to 2 weeks	7 day
2 weeks–1 month	21 days	2 weeks to 1 month	21 days
1–3 months	56 days	1 month to 3 months	56 days
3–6 months	126 days	3 months to 6 months	126 days
6 months–1 year	252 days	> 6 months	Unable to estimate
>1 year	Unable to estimate	‘not been told’	Unable to estimate
